# Approximate Minimum Selection with Unreliable Comparisons

**DOI:** 10.1007/s00453-021-00880-1

**Published:** 2021-11-01

**Authors:** Stefano Leucci, Chih-Hung Liu

**Affiliations:** 1 Department of Information Engineering, Computer Science and Mathematics, University of L’Aquila, L’Aquila, Italy; 2 Department of Computer Science, ETH Zürich, Zürich, Switzerland

**Keywords:** Approximate minimum selection, Unreliable comparisons, Independent errors

## Abstract

We consider the *approximate minimum selection* problem in presence of *independent random comparison faults*. This problem asks to select one of the smallest *k* elements in a linearly-ordered collection of *n* elements by only performing *unreliable* pairwise comparisons: whenever two elements are compared, there is a small probability that the wrong comparison outcome is observed. We design a randomized algorithm that solves this problem with a success probability of at least $1-q$ for $q \in (0, \frac{n-k}{n})$ and any $k \in [1, n-1]$ using $O\big ( \frac{n}{k} \big \lceil \log \frac{1}{q} \big \rceil \big )$ comparisons in expectation (if $k \ge n$ or $q \ge \frac{n-k}{n}$ the problem becomes trivial). Then, we prove that the expected number of comparisons needed by any algorithm that succeeds with probability at least $1-q$ must be ${\varOmega }(\frac{n}{k}\log \frac{1}{q})$ whenever *q* is bounded away from $\frac{n-k}{n}$, thus implying that the expected number of comparisons performed by our algorithm is asymptotically optimal in this range. Moreover, we show that the approximate minimum selection problem can be solved using $O( (\frac{n}{k} + \log \log \frac{1}{q}) \log \frac{1}{q})$ comparisons *in the worst case*, which is optimal when *q* is bounded away from $\frac{n-k}{n}$ and $k = O\big ( \frac{n}{\log \log \frac{1}{q}}\big )$.

## Introduction

In an ideal world, computational tasks are always carried out reliably, i.e., every operation performed by an algorithm behaves exactly as intended. Practical architectures, however, are error-prone and even basic operations can sometimes return the wrong results, especially when large-scale systems are involved. When dealing with these spurious results the first instinct is to try to detect and correct the errors as they manifest, so that the problems of interest can then be solved using classical (non fault-tolerant) algorithms. An alternative approach deliberately allows errors to interfere with the execution of an algorithm, in the hope that the computed solution will still be good, at least in an *approximate* sense. This begs the question: *is it possible to devise algorithms that cope with faults by design and return solutions that are demonstrably good?*

We investigate this question by considering a generalization of the fundamental problem of finding the minimum element in a totally-ordered set: in the *fault-tolerant approximate minimum selection* problem (${{\textsc {FT}}\hbox {-}{\textsc {Min}}} (k)$ for short) we wish to return one of the smallest *k* elements in a collection of size $n>k$ using only *unreliable pairwise comparisons*, i.e., comparisons in which the result can sometimes be incorrect due to *errors*. This allows, for example, to find a representative in the top percentile of the input set, or to obtain a good estimate of the minimum from a set of noisy observations.

In this paper we provide both upper and lower bounds on the number of comparisons needed by any (possibly randomized) algorithm that solves ${{\textsc {FT}}\hbox {-}{\textsc {Min}}} (k)$ with a success probability of at least $1-q$. Since for $q \ge \frac{n-k}{n}$ we can solve ${{\textsc {FT}}\hbox {-}{\textsc {Min}}} (k)$ by simply returning a random input element, we will focus on $q \in (0, q_{\mathrm{crit}})$, where $q_{\mathrm{crit}}= \frac{n-k}{n}$. We prove that ${{\textsc {FT}}\hbox {-}{\textsc {Min}}} (k)$ can be solved using $O\big ( \frac{n}{k} \log \frac{1}{q}\big )$ comparisons *in expectation*,^1^ and that this number of comparisons is asymptotically optimal when *q* is *bounded away* from $q_{\mathrm{crit}}$. Moreover, we show that whenever $k = O(\frac{n}{\log \log \frac{1}{q}})$ we can use the same asymptotic number of comparisons also *in the worst case*.

Our results have applications in any setting that is subject to random comparison errors (e.g., due to communication interferences, alpha particles, charge collection, cosmic rays [4, 8], or energy-efficient architectures where the energy consumed by the computation can be substantially reduced if a small fraction of faulty results is allowed [2, 9, 10, 29]), or in which performing accurate comparisons is too resource-consuming (think, e.g., of the elements as references to remotely stored records) while approximate comparisons can be carried out much quicker. One concrete application might be selecting one of the top-tier products from a collection of items built using an imprecise manufacturing process (i.e., a high-quality cask from a distillery or a fast semiconductor from a fabrication facility). In these settings, products can be compared to one another (either by human experts or by automated tests), yet the result of the comparisons are not necessarily accurate.^2^

Before presenting our results in more detail, we briefly discuss the considered error model.

### The Error Model

We consider *independent random comparison faults*, a simple and natural error model, in which there exists a *true* strict ordering relation among the set *S* of *n* input elements, yet algorithms are only allowed to gather information on this relation via *unreliable* comparisons between pairs of elements. The outcome of a comparison involving two distinct elements *x* and *y* can be either “<” or “>” to signify that *x* is reported as “smaller than” or “larger than” *y*, respectively. Most of the times the outcome of a comparison will correspond to the true relative order of the compared elements, but there is a probability upper bounded by a constant $p < \frac{1}{2}$ that the wrong result will be observed instead. An algorithm can compare the same pair of elements more than once and, when this happens, the outcome of each comparison is chosen *independently* of the previous results. In a similar way, comparisons involving different pairs of elements are also assumed to be independent.

The above error model was first considered in the 80s and 90s when the related problems of finding the minimum, selecting the *k*-th smallest element, and of sorting a sequence have been studied [14, 30, 31]. The best solutions to these problems are due to Feige et al. [14], who provided Monte Carlo algorithms having a success probability of $1-q$ and requiring $O\big (n \log \frac{1}{q}\big )$, $O\big (n\log \frac{\min \{k,n-k\}}{q}\big )$, and $O\big (n\log \frac{n}{q}\big )$ comparisons in the worst case, respectively. Moreover, the authors also provide matching lower bounds, thus showing that the above algorithms use the asymptotically optimal number of comparisons in the worst case. In the sequel we will invoke the minimum finding algorithm of [14]—which we name *FindMin*—as a subroutine. We therefore find convenient to restate the following theorem from [14] using our notation:

#### Theorem 1

([14, Theorem 3.5]) Given a set *S* of *n* elements and a parameter $\varrho \in (0, \frac{1}{2})$, algorithm *FindMin* performs $O\left( n \log \frac{1}{\varrho }\right)$ comparisons in the worst case and returns the minimum of *S* with a success probability of at least $1-\varrho$.

### Our Contributions

We design a randomized algorithm that solves ${{\textsc {FT}}\hbox {-}{\textsc {Min}}} (k)$ with a success probability of at least $1-q$ using $O(\frac{n}{k} \log \frac{1}{q})$ comparisons in expectation, where $q \in (0, q_{\mathrm{crit}})$. Moreover, we show that the expected number of comparisons performed by our algorithm is asymptotically optimal when *q* is bounded away from $q_{\mathrm{crit}}$ by proving that any algorithm that succeeds with probability at least $1-q$ requires ${\varOmega }(\frac{n}{k}\log \frac{1}{q})$ comparisons in expectation.

We also show how to additionally guarantee that the *worst-case* number of comparisons required by our algorithm will be $O( (\frac{n}{k} + \log \log \frac{1}{q}) \cdot \log \frac{1}{q} )$. This implies that, as soon as $k = O\big (\frac{n}{\log \log \frac{1}{q}}\big )$, we can solve ${{\textsc {FT}}\hbox {-}{\textsc {Min}}} (k)$ with a success probability of at least $1-q$ using $O(\frac{n}{k}\log \frac{1}{q})$ comparisons, which is asymptotically optimal when *q* is bounded away from $q_{\mathrm{crit}}$.

A possible way to evaluate different algorithms for ${{\textsc {FT}}\hbox {-}{\textsc {Min}}} (k)$ is that of comparing the range of values of *k* that they are able to handle if we impose an (asymptotic) upper limit of *T* on the (possibly expected) number of comparisons that they are allowed to perform. For example, if we require the algorithms to succeed *with high probability* (w.h.p., i.e., for $q=\frac{1}{n}$) and pick $T={\varTheta }(n)$, the natural algorithm that executes *FindMin* with $\varrho =O(\frac{1}{n})$ on a randomly chosen subset of $O(\frac{n}{k}\log n)$ elements only works for $k = {\varOmega }( \log ^2 n )$. For the same choice of *T* and *q* our algorithm works for any $k={\varOmega }(\log n)$, thus exhibiting a quadratic difference w.r.t. the smallest achievable values of *k*. When $T=\omega (\log n) \cap o(\log ^2 n)$, the natural algorithm cannot provide any (non-trivial) guarantee on the rank of the returned element w.h.p., while our algorithm works for any $k={\varOmega }(\frac{n}{T}\log n)$. To summarize, our algorithm is able to handle the asymptotically optimal range of *k* if (i) *T* refers to an expected number of comparisons, or (ii) *T* refers to a worst-case number of comparisons and $T = \omega (\log n \cdot \log \log n)$.

### Our Techniques

To obtain our positive results we start by designing a reduction that transforms an instance of ${{\textsc {FT}}\hbox {-}{\textsc {Min}}} (k)$ into an instance of ${{\textsc {FT}}\hbox {-}{\textsc {Min}}} \left( \frac{3}{4}n\right)$ with $n={\varTheta }(\log \frac{1}{q})$ elements. This reduction shows that if it is possible to solve ${{\textsc {FT}}\hbox {-}{\textsc {Min}}} \big (\frac{3}{4}n\big )$ with a success probability of at least $1-\frac{q}{2}$ using *T*(*n*) comparisons, then ${{\textsc {FT}}\hbox {-}{\textsc {Min}}} (k)$ can be solved with a success probability of at least $1-q$ using $O\big (\frac{n}{k}\log \frac{1}{q} \big ) +T\left( {\varTheta }(\log \frac{1}{q}) \right)$ comparisons. This allows us to focus on solving ${{\textsc {FT}}\hbox {-}{\textsc {Min}}} \left( \frac{3}{4}n\right)$ with a success probability of at least $1-q$ using $O(\frac{1}{q})$ comparisons.

We do so using a *noisy oracle* that is able to detect, using a constant number of comparisons, whether an element is among the smallest elements in *S* with an error probability upper bounded by a (small) constant. We employ this noisy oracle in an iterative algorithm that considers one random input element at a time: whenever $x \in S$ is considered, *x* is tested by querying the oracle multiple times, and it is returned only if most of the query answers report *x* as one smallest elements in *S*. The amount of queries performed during each test increases with the iteration number and is chosen to simultaneously ensure that (i) the overall expected number of comparisons is $O(\frac{1}{q})$, and (ii) the probability of (wrongly) returning an element that is too large is at most *q*. Using our reduction, the above algorithm can be immediately transformed into an algorithm that solves ${{\textsc {FT}}\hbox {-}{\textsc {Min}}} (k)$ using $O(\frac{n}{k} \log \frac{1}{q})$ comparisons in expectation and $O(\frac{n}{k} \log \frac{1}{q} + \log ^2 \frac{1}{q})$ comparisons in the worst case.

To reduce the number of comparisons needed in the worst case, we design an algorithm for ${{\textsc {FT}}\hbox {-}{\textsc {Min}}} \big (\frac{3}{4}n\big )$ that is reminiscent of knockout-style tournaments and always performs $O(n \log n + n \log \frac{1}{q} + n^{-1} \log ^2 \frac{1}{q})$ comparisons. Thanks to our reduction, this latter algorithm improves the worst-case number of comparisons required to solve ${{\textsc {FT}}\hbox {-}{\textsc {Min}}} (k)$ to $O\big (\frac{n}{k}\log n + (\log \frac{1}{q})\log \log \frac{1}{q}\big )$, which is optimal for $k = O\big ( \frac{n}{\log \log \frac{1}{q}} \big )$ and *q* bounded away from $q_{\mathrm{crit}}$.

Regarding our negative results, we obtain our lower bound of ${\varOmega }(\frac{n}{k} \log \frac{1}{q})$ using three different strategies depending on the values of *k*, *n*, and *q* (where *q* is bounded away from $q_{\mathrm{crit}}$). For $n \ge 2k$ and $q \le \frac{1}{4}$, which we deem the most interesting case, we reduce ${{\textsc {FT}}\hbox {-}{\textsc {Min}}} (k)$ to ${{\textsc {FT}}\hbox {-}{\textsc {Min}}} (1)$ so that (an extension of) the lower bound of [14] applies. For $k< n < 2k$ and $q \le \frac{1}{4}$, we construct a set ${\mathcal {I}}$ of roughly $\frac{n}{n-k}$ instances of *n* elements each such that the generic *i*-th input position contains one of the largest $n-k$ elements in at least one *bad* instance in ${\mathcal {I}}$. In order to return the *i*-th input element, an algorithm for ${{\textsc {FT}}\hbox {-}{\textsc {Min}}} (k)$ needs to detect whether the input instance is *bad* w.r.t. *i* with a sufficiently high confidence, which requires ${\varOmega }(\log \frac{1}{q})$ comparisons in expectation. Finally, for $q > \frac{1}{4}$, we show how to improve the success probability of any algorithm that solves ${{\textsc {FT}}\hbox {-}{\textsc {Min}}} (k)$ from $1-q < \frac{3}{4}$ to at least $\frac{3}{4}$ with only a small blow-up in the number of comparisons, allowing us to employ one of the two lower bound strategies described above.

### Other Related Works

The problem of finding the *exact* minimum of a collection of elements using unreliable comparisons had already received attention back in 1987 when Ravikumar et al. [32] considered the variant in which only up to *f* comparisons can fail and proved that ${\varTheta }(f n)$ comparisons are needed in the worst case. Notice that, in our setting with *q* bounded away from $q_{\mathrm{crit}}$, $f= {\varOmega }(\frac{n}{k} \log \frac{1}{q})$ in expectation since ${\varOmega }(\frac{n}{k} \log \frac{1}{q})$ comparisons are necessary (as we show in Sect. 6) and each comparison fails with constant probability. In [1], Aigner considered a *prefix-bounded* probability of error $p < \frac{1}{2}$: at any point during the execution of an algorithm, at most a *p*-fraction of the past comparisons could have failed. Here, the situation significantly worsens as up to ${\varTheta }(\frac{1}{1-p})^n$ comparisons might be necessary to find the minimum (and this is tight). Moreover, if the fraction of erroneous comparisons is *globally* bounded by $\rho$, and $\rho ={\varOmega }(\frac{1}{n})$, then Aigner also proved that no algorithm can succeed with certainty [1]. The landscape improves when we assume that errors occur independently at random: in addition to the already-cited algorithm by Feige et al. [14] (see Sect. 1.1), a recent paper by Braverman et al. [6] also considered the *round complexity* and the number of comparisons needed by partition and selection algorithms. The results in [6] imply that, for constant error probabilities, ${\varTheta }(n \log n)$ comparisons are needed by any algorithm that selects the minimum w.h.p.

Recently, Chen et al. [11] focused on computing the smallest *k* elements given *r* independent noisy comparisons between each pair of elements. For this problem, in a more general error model, they provide a tight algorithm that requires at most $O(\sqrt{n} \,polylog \, n)$ times as many samples as the best possible algorithm that achieves the same success probability.

If we turn our attention to the related problem of sorting with faults, then ${\varOmega }(n \log n + fn)$ comparisons are needed to correctly sort *n* elements when up to *f* comparisons can return the wrong answer, and this is tight [3, 23, 26]. In the prefix-bounded model, the result in [1] on minimum selection also implies that $(\frac{1}{1-p})^{O(n \log n)}$ comparisons are sufficient for sorting, while a lower bound of ${\varOmega }\big ( ( \frac{1}{1-p} )^n \big )$ holds even for the easier problem of checking whether the input elements are already sorted [5]. The problem of sorting when faults are permanent (or, equivalently, when a pair of elements can only be compared once) has also been extensively studied and it exhibits connections to both the *rank aggregation* problem and to the *minimum feedback arc set* [6, 7, 16–18, 20–22, 24, 27].

Another related family of problems falls in the framework of Rényi–Ulam games: in these two-player games a *responder* secretly selects an object *x* from a known universe *U* and a *questioner* needs to identify *x* by asking up to *n* questions to the responder. The responder can lie up to *f* times and wins if the questioner fails to uniquely identify *x*. As an example, if $U = \{1, \dots , m\}$ and the questions are comparisons the of form “Is $x \le c$?”, where $c \in U$ is chosen by the questioner, then the questioner can always win by asking at most $\log m + f \log \log n + O(f \log f)$ questions, which is tight [33].^3^ More broadly, Rényi–Ulam games and other search games have been extensively studied for a wide variety of search spaces and question types, as discussed in [31].

Other error-prone models have also been considered in the context of optimization algorithms [19] and in the design resilient data strictures [15, 25]. For more related problems on the aforementioned and other fault models, we refer the interested reader to [31] for a survey and to [12] for a monograph.

Finally, we point out that, in the fault-free case, a simple sampling strategy allows to find one of the smallest *k* elements with probability at least $1-q$ using $O(\min \{n, \frac{n}{k} \log \frac{1}{q} \})$ comparisons.

### Paper Organization

In Sect. 2 we give some preliminary remarks and we outline a simple strategy to reduce the error probability. Section 3 describes our reduction from ${{\textsc {FT}}\hbox {-}{\textsc {Min}}} (k)$ to ${{\textsc {FT}}\hbox {-}{\textsc {Min}}} (\frac{3}{4}n)$. In Sects. 4 and  5 we design two algorithms that solve ${{\textsc {FT}}\hbox {-}{\textsc {Min}}} (k)$ using $O(\frac{n}{k} \log \frac{1}{q})$ comparisons in expectation and $O(\frac{n}{k} \log \frac{1}{q} + (\log \frac{1}{q}) \log \log \frac{1}{q})$ comparisons in the worst case, respectively. Finally, Sect. 6 is devoted to proving our lower bounds.

## Preliminaries

We will often draw elements from the input set into one or more (multi)sets using sampling with replacement, i.e., we allow multiple copies of an element to appear in the same multiset. We will then perform comparisons among the elements of these multisets as if they were all distinct: when two copies of the same element are compared, we break the tie using any arbitrary (but consistent) ordering among the copies.

According to our error model, each comparison fault happens independently at random with probability at most $p \in (0, \frac{1}{2})$. This error probability can be reduced by repeating a comparison multiple times using a simple majority strategy. The same strategy also works in the related setting in which pairwise comparisons are no longer allowed but we have access to a *noisy oracle* ${\mathcal {O}}$ that can be queried with an element $x \in S$ and returns either true or false. In this setting each $x \in S$ is associated with a correct binary answer and, when ${\mathcal {O}}$ is queried with *x*, it returns the correct answer with probability at least $1-p > \frac{1}{2}$ (and the wrong answer with the complementary probability). The errors in ${\mathcal {O}}$’s answers are independent. Next lemma provides a lower bound on the probability of correctness of the majority strategy:

### Lemma 1

Let *x* and *y* be two distinct elements. For any error probability upper bounded by a constant $p \in [0, \frac{1}{2} )$ there exists a constant $c_p \in {\mathbb {N}}^+$ such that the strategy that compares *x* and *y* (resp. queries ${\mathcal {O}}$ with *x*) $2 c_p \cdot t+1$ times and returns the majority result is correct with probability at least $1- e^{-t}$.

### Proof

Suppose, w.l.o.g., that $x < y$. Let $X_i \in \{0,1\}$ be an indicator random variable that is 1 iff the *i*-th comparison (resp. query result) is correct. Since the $X_i$s are independent Bernoulli random variables with parameter at least $1-p$, $\sum _{i=1}^{2 c_p \cdot t+1} X_i$ *stochastically dominates* [28, Definition 17.1] a binomial random variable *X* with parameters $2 \eta = 2 c_p t +1$ and $1-p$,^4^ and hence ${\mathbb {E}}[X]=2\eta (1-p)=(1-p)(2c_p\cdot t+1)$. Moreover, since $p < 1/2$, we know that $2(1-p) > 1$ and hence we can use the Chernoff bound $\Pr \left( X \le (1-\delta ){\mathbb {E}}[X] \right) \le \exp \left( -\frac{\delta ^2 {\mathbb {E}}[X]}{2} \right) ,\; \forall \delta \in (0,1)$ to upper bound the probability of failure of the majority strategy (see [28, Theorem 4.5 (2)]). Indeed:

$$\begin{aligned} \Pr (X \le \eta )&= \Pr \left( X \le \frac{1}{2(1-p)} {\mathbb {E}}[X] \right) \le \exp \left( - \frac{ (2 (1-p) - 1)^2}{8(1-p)^2} 2\eta (1-p) \right) \\&= \exp \left( - \frac{(1-2p)^2}{4(1-p)} \eta \right) < \exp \left( - c_p t \frac{(1-2p)^2}{4(1-p)} \right) , \end{aligned}$$

which satisfies claim once we choose $c_p = \left\lceil \frac{4(1-p)}{(1-2p)^2} \right\rceil$. $\square$

## Our Reduction

In this section we reduce the problem of solving ${{\textsc {FT}}\hbox {-}{\textsc {Min}}} (k)$ to the problem of solving ${{\textsc {FT}}\hbox {-}{\textsc {Min}}} (\frac{3}{4}n)$.^5^ We will say that an element *x* is *small* if it is one of the smallest *k* elements of *S*, otherwise we say that *x* is *large*. The reduction constructs a set $S^*$ of size *m* that contains at least $\frac{3}{4}m$ small elements, where the value of *m* will be determined later. The set $S^*$ is selected as follows:

- Create *m* sets by independently sampling, with replacement, $\lceil 3 \frac{n}{k} \rceil$ elements per set from *S*.
- Run *FindMin* (Theorem 1) with failure probability $\varrho = \frac{1}{10}$ on each of the sets. Let $S^* = \{x_1, \dots , x_m\}$ be the collection containing the returned elements, where $x_i$ is the element returned by the execution of *FindMin* on the *i*-th set.

Using Theorem 1, Lemma 1 and the Chernoff bound, we are able to prove the following lemma:

### Lemma 2

The probability that fewer than $\frac{3}{4}m$ elements in $S^*$ are small is at most $e^{- \frac{m}{240}}$.

### Proof

Since the *i*-th set contains at least $3 \frac{n}{k}$ elements and each of them is independently small with a probability of $\frac{k}{n}$, the probability that no element in the *i*-th set is small is upper bounded by:

$$\begin{aligned} \left( 1- \frac{k}{n}\right) ^{3 \frac{n}{k}} \le \left( e^{-\frac{k}{n}} \right) ^{3 \frac{n}{k}} = e^{-3} < \frac{1}{20}, \end{aligned}$$

where we used the inequality $1+r \le e^r$ for $r \in {\mathbb {R}}$. In other words, for every *i*, the event “the *i*-th set contains a small element” has probability at least $1-\frac{1}{20}$. Moreover, since we chose $\varrho =\frac{1}{10}$, the probability that *FindMin* returns the correct minimum of the *i*-th set is at least $1-\frac{1}{10}$ (see Theorem 1). Clearly, if both of the previous events happen, $x_i$ must be a small element, and by the union bound, the complementary probability is at most $\frac{1}{20} + \frac{1}{10} < \frac{1}{6}$.

Let $X_i$ be an indicator random variable that is 1 iff $x_i$ is a small element so that $X = \sum _{i=1}^m X_i$ is the number of small elements in $S^*$. Since the $x_i$s are independently small with a probability of at least $\frac{5}{6}$, the variable *X* stochastically dominates a Binomial random variable with parameters *m* and $\frac{5}{6}$. As a consequence ${\mathbb {E}}[X] \ge \frac{5}{6} m$ and, by using the Chernoff bound [28, Theorem 4.5 (2)], we obtain:

$$\begin{aligned} \Pr \left( X \le \frac{3}{4} m \right) \le \Pr \left( X \le \frac{9}{10} {\mathbb {E}}[X]\right) \le e^{- \frac{1}{2}(\frac{1}{10})^2 \cdot \frac{5}{6} m} =e^{- \frac{m}{240}}. \end{aligned}$$

$$\square $$

We are now ready to show the consequence of the above reduction:

### Lemma 3

Let *A* be an algorithm that solves ${{\textsc {FT}}\hbox {-}{\textsc {Min}}} ( \frac{3}{4}n )$ with a success probability of at least $1-q_A \in (\frac{1}{2}, 1)$ using at most $T(n, q_A)$ comparisons in the worst case (resp. in expectation), for any choice of $q_A \in (0, \frac{1}{2})$. For any *k* and any $q \in (0, 1)$, there exists an algorithm that solves ${{\textsc {FT}}\hbox {-}{\textsc {Min}}} (k)$ with a success probability of at least $1-q$ using $O\left( \frac{n}{k} \log \frac{1}{q} \right) + T\left( {\varTheta }(\log \frac{1}{q}), \frac{q}{2} \right)$ comparisons in the worst case (resp. in expectation).

### Proof

We first choose $m = \big \lceil 240 \ln \frac{2}{q} \big \rceil$ and we compute the set $S^*$ according to our reduction. Then we run *A* on $S^*$ with a failure probability of $q_A = \frac{q}{2}$, and we answer with the element it returns. Notice that first step of the reduction requires no comparisons. Moreover, since each of the $m = O(\log \frac{1}{q})$ executions of *FindMin* requires $O( \frac{n}{k} \log \frac{1}{\varrho }) = O(\frac{n}{k})$ comparisons (see Theorem 1 and recall that $\varrho =1/10$), the worst-case number of comparisons performed during the second step is $O(\frac{n}{k} \log \frac{1}{q})$. Overall, the total number of comparisons is $O\left( \frac{n}{k} \log \frac{1}{q} \right) + T\left( {\varTheta }(\log \frac{1}{q}), \frac{q}{2} \right)$ as claimed. This upper bound holds in the worst-case if $T(n, q_A)$ refers to a worst-case number of comparisons and in expectation if $T(n, q_A)$ refers to an expected number of comparisons.

We now consider the probability of success. By Lemma 2, the probability that fewer than $\frac{3}{4}m$ elements in $S^*$ are small is at most $e^{-\frac{m}{240}} \le e^{- \ln \frac{2}{q}} = \frac{q}{2}$. Since the probability that *A* fails to return one of the smallest $\frac{3}{4}m$ elements in $S^*$ is at most $q_A = \frac{q}{2}$, the claim follows by using the union bound. $\square$

It is not hard to see that, if we choose algorithm *A* in Lemma 3 to be *FindMin*, we have $T(n, q_A)= O(n\log \frac{1}{q_A})$ which, thanks to our reduction, allows us to solve ${{\textsc {FT}}\hbox {-}{\textsc {Min}}} (k)$ with a success probability of at least $1 - q$ using $O(\frac{n}{k}\log \frac{1}{q} +\log ^2 \frac{1}{q})$ comparisons. This number of comparisons matches our lower bound of ${\varOmega }(\frac{n}{k}\log \frac{1}{q})$ (see Theorem 5 in Sect. 6) when $k = O\big (\frac{n}{\log \frac{1}{q}}\big )$ and *q* is bounded away from $q_{\mathrm{crit}}$. Nevertheless, the major difficulty in solving ${{\textsc {FT}}\hbox {-}{\textsc {Min}}} (k)$ lies in the case $k=\omega \big (\frac{n}{\log \frac{1}{q}}\big )$.

## Solving ${{\textsc {FT}}\hbox {-}{\textsc {Min}}} (k)$ Using the Asymptotically Optimal Expected Number of Comparisons

In this section, we will solve ${{\textsc {FT}}\hbox {-}{\textsc {Min}}} (k)$ with a success probability of at least $1-q$ using $O(\frac{n}{k} \log \frac{1}{q})$ comparisons in expectation. By Lemma 3, it is sufficient to devise an algorithm that solves ${{\textsc {FT}}\hbox {-}{\textsc {Min}}} (\frac{3}{4}n)$ with a success probability of at least $1-q$ using $O(\log \frac{1}{q})$ comparisons in expectation. We assume that $n \ge 4$ since otherwise we can simply return an element selected uniformly at random from *S*.

In designing such an algorithm we will use a *noisy oracle* ${\mathcal {O}}$ that can be queried with an element $x \in S$ and provides a *guess* on whether *x* is *small*, i.e., among the smallest $\left\lceil \frac{3}{4}n\right\rceil$ elements of *S*, or *large* (i.e., among the largest $\left\lfloor \frac{1}{4}n\right\rfloor$ elements of *S*). More precisely, for $\delta \in [0,1]$, let $S^-_\delta$ denote the set containing the smallest $\lceil \delta n \rceil$ elements of *S*, and let $S^+_\delta$ denote $S \setminus S^-_\delta$. Then, ${\mathcal {O}}$ satisfies the following conditions:

- ${\mathcal {O}}$ reports an element *x* to be small with probability at least $1 - \frac{2}{5}$ if $x \in S^-_{1/3}$ and with probability at most $\frac{2}{5}$ if $x \in S^+_{3/4}$. In other words, ${\mathcal {O}}$ identifies whether an element in $S^-_{1/3} \cup S^+_{3/4}$ is small or large with a failure probability of at most $\frac{2}{5}$;
- Queries to ${\mathcal {O}}$ can be repeated and errors in the answers are independent;
- Each query to ${\mathcal {O}}$ is implemented using a constant number of comparisons between elements in *S*.

Notice that ${\mathcal {O}}$ provides no guarantees on the accuracy of its answers when $x \in S^-_{3/4} \setminus S^-_{1/3}$. We will show how to build such an oracle in Sect. 4.1.

Let $c_{\mathcal {O}}$ be the constant of Lemma 1 for $p=\frac{2}{5}$. Our algorithm works in phases: in the generic *i*-th phase we select one element $x_i$ uniformly at random from *S*, and we perform a *test* on $x_i$. This test consists of $2 c_{\mathcal {O}} \Big \lceil \ln \frac{2^i}{q} \Big \rceil + 1$ queries to ${\mathcal {O}}$, and it *succeeds* if *x* is reported as small by the majority of the queries (otherwise it *fails*). If the test on $x_i$ succeeds we return $x_i$. Otherwise we move to the next phase. We name the above algorithm *GeometricTest* since the probability that a test succeeds when a large element is considered decreases geometrically w.r.t. the phase number, as we will show in Sect. 4.2.

### Implementing ${\mathcal {O}}$

To describe our implementation of ${\mathcal {O}}$ we can assume, without loss of generality, that $p \le \frac{1}{16}$ (if $p > \frac{1}{16}$, we can simulate each comparison by returning the majority result of $6c_p+1$ comparisons, as shown by Lemma 1). The oracle ${\mathcal {O}}$ answers a query for an element $x \in S'$ by comparing *x* with a randomly sampled element *y* from $S \setminus \{x\}$. If *x* compares smaller than *y*, then *x* is reported as small, otherwise it is reported as large. Suppose that $x \in S^-_{1/3}$, if *x* is (incorrectly) reported as large at least one of the following two conditions must be true (i) $y \in S^-_{1/3}$ or (ii) the comparison between *x* and *y* returned the wrong result. The first condition is true with probability at most $\frac{\lceil n/3 \rceil - 1}{n-1} \le \frac{1}{3}$ while the second condition is true with probability at most $p \le \frac{1}{16}$. Therefore the probability that *x* is reported as large is at most $\frac{1}{3} + \frac{1}{16} < \frac{2}{5}$. If $x \in S^+_{3/4}$ then, in order for *x* to be (incorrectly) reported as small, we must have that (i) $y \in S^+_{3/4}$ or (ii) the comparison between *x* and *y* returned the wrong result. The first condition is true with probability at most $\frac{n - \lceil 3n/4 \rceil - 1}{n-1} \le \frac{1}{4}$ while the second condition is true with probability at most $p \le \frac{1}{16}$. Overall, *x* is reported as small with probability at most $\frac{1}{4} + \frac{1}{16} < \frac{2}{5}$.

### Analysis of the Expected Number of Comparison and of the Success Probability of *GeometricTest*

The following lemmas respectively provide an upper bound on the expected number of comparisons and a lower bound on the success probability of *GeometricTest*.

#### Lemma 4

*GeometricTest* performs $O(\log \frac{1}{q})$ comparisons in expectation.

#### Proof

Consider a generic phase *i*. Assuming that the algorithm did not stop during phases $1, 2, \dots , i-1$, the probability that it stops during phase *i* is at least:

$$\begin{aligned} \Pr \left( x_i \in S^-_{\frac{1}{3}}\right) \cdot \Pr \left( \text {the test on}\, x_i \,\text {succeeds} \, \Big | \, x_i \in S^-_{\frac{1}{3}}\right) \ge \frac{1}{3} \cdot \left( 1 - \frac{q}{2^i} \right) \ge \frac{1}{6}, \end{aligned}$$

where we used the fact that a test on an element from $S^-_{\frac{1}{3}}$ performed during the *i*-th phase succeeds with probability at least $1 - e^{- \big \lceil \ln \frac{2^i}{q} \big \rceil } \ge 1 - \frac{q}{2^i} \ge \frac{1}{2}$, as shown by Lemma 1. Then, the number of phases executed by the algorithm is stochastically dominated by a *geometric random variable* *X* with parameter $\frac{1}{6}$ (see [28, Definition 2.8]).

Since, for some constant $\kappa >0$, at most $\kappa \ln \frac{2^i}{q}$ comparisons are performed during phase *i*,^6^ we have that the overall number $C_i$ of comparisons performed during phases $1, \dots , i$ is upper bounded by $\sum _{j=1}^i \kappa \ln \frac{2^j}{q} \le \kappa \log \frac{2}{q} \cdot \sum _{j=1}^i j \le i^2 \kappa \log \frac{2}{q}$. Then, the expected number of comparisons performed by *GeometricTest* is at most:

$$\begin{aligned} \sum _{i=1}^\infty \Pr (X = i) C_i&\le \sum _{i=1}^\infty \frac{1}{6} \left( \frac{5}{6} \right) ^{i-1} i^2 \kappa \log \frac{2}{q} \\&= \frac{\kappa }{5} \log \frac{2}{q} \cdot \sum _{i=1}^\infty i^2 \left( \frac{5}{6} \right) ^i = 66\kappa \log \frac{2}{q} = O\left( \log \frac{1}{q} \right) , \end{aligned}$$

where we used the equality $\sum _{i=1}^\infty i^2 (5/6)^i = 330$ which follows from the more general identity $\sum _{i=1}^\infty i^2 r^i = \frac{r^2 + r}{(1-r)^3}$ for $r \in (0,1)$ [13]. $\square$

#### Lemma 5

*GeometricTest* solves ${{\textsc {FT}}\hbox {-}{\textsc {Min}}} (\frac{3}{4}n)$ with a success probability of at least $1-\frac{q}{4}$.

#### Proof

If *GeometricTest* fails, then either it does not terminate or it returns an element in $S^+_{3/4}$. Since it is easy to see that the algorithm terminates almost surely,^7^ we can focus on upper bounding the probability $\rho _i$ that the algorithm terminates at the end of a generic phase *i* by returning an element in $S^+_{3/4}$. In order for this to happen we must have that (i) $x_i$ is large and (ii) was reported as small by at least $c_{\mathcal {O}} \Big \lceil \ln \frac{2^i}{q} \Big \rceil + 1$ of the $2 c_{\mathcal {O}} \Big \lceil \ln \frac{2^i}{q} \Big \rceil + 1$ queries to ${\mathcal {O}}$. The probability of (i) is at most $\frac{1}{4}$, and by Lemma 1, the probability of (ii) given (i) is at most $e^{-\big \lceil \ln \frac{2^i}{q} \big \rceil } \le \frac{q}{2^i}$, implying that $\rho _i \le \frac{1}{4}\cdot \frac{q}{2^i}=\frac{q}{2^{i+2}}$. We can now use the union bound over the different phases to upper bound the overall failure probability with $\sum _{i=1}^\infty \rho _i \le \sum _{i=1}^\infty \frac{q}{2^{i+2}} = \frac{q}{4}$. $\square$

Combining Lemmas 4 and 5 we can conclude that *GeometricTest* solves ${{\textsc {FT}}\hbox {-}{\textsc {Min}}} (\frac{3}{4}n)$ with a success probability of at least $1-q$ using $O(\log \frac{1}{q})$ comparisons in expectation. Lemma 3 immediately implies the following theorem:

#### Theorem 2

${{\textsc {FT}}\hbox {-}{\textsc {Min}}} \left( k \right)$ can be be solved with a success probability of at least $1-q$ using $O( \frac{n}{k} \log \frac{1}{q} )$ comparisons in expectation.

We conclude this section by pointing out that, since each phase of *GeometricTest* has a probability of at least $\frac{1}{6}$ of returning a small element (as shown in the proof of Lemma 4), we can consider the variant obtained by running *GeometricTest* for up to $8 \big \lceil \log \frac{1}{q} \big \rceil$ phases (if the number of phases is exceeded we return a random element). We name this variant *TruncatedGeometricTest*. We can upper bound the worst-case number of comparisons performed by *TruncatedGeometricTest* with $O\big ( \sum _{i=1}^{8\lceil \log 1/q \rceil } \log \frac{2^i}{q}\big ) = O(\log ^2 \frac{1}{q})$ and lower bound the success probability with $1 - \frac{q}{4} - \left( \frac{5}{6} \right) ^{8 \lceil \log \frac{1}{q} \rceil } \ge 1 - \frac{q}{4} - \left( \frac{5}{6} \right) ^4 \cdot \left( \frac{5}{6} \right) ^{4 \log \frac{1}{q}}> 1 - \frac{q}{4} - \frac{q}{2} > 1 - q$. Combining *TruncatedGeometricTest* with Lemma 3, we obtain an algorithm that solves ${{\textsc {FT}}\hbox {-}{\textsc {Min}}} \left( k \right)$ with a success probability of at least $1-q$ using $O( \frac{n}{k} \log \frac{1}{q} )$ comparisons in expectation and $O(\frac{n}{k} \log \frac{1}{q} + \log ^2 \frac{1}{q})$ comparisons in the worst case. This latter algorithm uses the same worst-case number of comparisons as the one that can obtained by combining *FindMin* with Lemma 3, but it uses fewer comparisons in expectation (see the discussion at the end Sect. 3 for details). In the next section we will show how to reduce the asymptotic number of comparisons needed in the worst case.

## Solving ${{\textsc {FT}}\hbox {-}{\textsc {Min}}} (k)$ Using an Almost-Optimal Number of Comparisons in the Worst Case

In this section, we solve ${{\textsc {FT}}\hbox {-}{\textsc {Min}}} (k)$ with a success probability of at least $1-q$ using $O\big ((\frac{n}{k}+ \log \log \frac{1}{q}) \log \frac{1}{q}\big )$ comparisons in the worst case. For the sake of simplicity, we assume that *n* is a power of two.^8^ We let $\rho \in (0, \frac{1}{2}]$ be a parameter that will be chosen later, and we design an algorithm that requires $O( n \cdot \log \frac{1}{\rho } \cdot (\log n + \log \frac{1}{\rho }))$ comparisons to solve ${{\textsc {FT}}\hbox {-}{\textsc {Min}}} \big (\frac{3}{4}n \big )$ with a success probability of at least $1 - \rho ^n$.

**Fig. 1:**
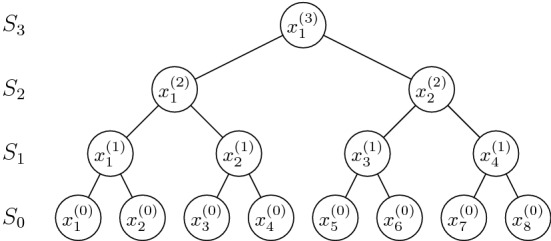
An example of the complete binary tree representing an execution of our algorithm when the input sequence *S* contains $n=8$ elements. Each internal vertex $x^{(i)}_j$ is the winner of a match (consisting of $2 c_p \big \lceil 2^{i} \ln \frac{1}{\rho } \big \rceil + 3$ comparisons) between its two children $x^{(i-1)}_{2j-1}$ and $x^{(i-1)}_{2j}$

Our algorithm simulates a knockout tournament and works in $\log n$ rounds. In the beginning we construct a set $S_0 = \{ x_1^{(0)}, x_2^{(0)}, \dots , x_n^{(0)} \}$ containing *n* elements from *S*, where each $x_j^{(0)}$ is obtained by running *FindMin* with a failure probability of $\varrho = \frac{\rho ^2}{2}$ on a (multi)set $X_j$ of $\big \lceil 2 \log \frac{1}{\rho } \big \rceil$ elements randomly sampled *with replacement* from *S*. Then, in the generic *i*-th round we match together $\frac{|S_{i-1}|}{2} = \frac{n}{2^{i}}$ pairs of elements from the set $S_{i-1}$, and we add the *match winners* to a new set $S_i = \{ x_1^{(i)}, x_2^{(i)}, \dots , x_{n/2^i}^{(i)} \} \subset S_{i-1}$. Specifically, for each $j=1,\dots ,\frac{n}{2^{i}}$ we run a match between $x^{(i-1)}_{2j-1}$ and $x^{(i-1)}_{2j}$ consisting of $2 c_p \big \lceil 2^{i} \ln \frac{1}{\rho } \big \rceil + 3$ comparisons, where $c_p$ is the constant of Lemma 1. The winner $x_j^{(i)}$ of the match is the element that is reported to be smaller by the majority of the comparisons.

After the $(\log n)$-th round we are left with a set $S_{\log n}$ containing a single element: this element is *winner of the tournament*, i.e., it is the element returned by our algorithm. The above algorithm can be visualized as a complete binary tree of height $\log n$ in which the leaves are the elements in $S_0$, the root is the unique element in $S_{\log n}$, and each internal vertex at depth $\log n - i$ represents some element $x^{(i)}_j \in S_i$ having $x^{(i-1)}_{2j-1}$ and $x^{(i-1)}_{2j}$ as its two children. See Fig. 1 for an example.

As in the previous section, we will say that an element is *small* if it is among the $\left\lceil \frac{3}{4}n \right\rceil$ smallest element of *S*, and *large* otherwise. The following lemma provides a lower bound on the success probability of our algorithm:

### Lemma 6

Consider a tournament among *n* elements, where *n* is a power of 2. The probability that the winner of the tournament is a small element is at least $1 - \rho ^{n+1}$.

### Proof

We prove by induction on $i=0,\dots ,\log n$ that $\forall x^{(i)}_j \in S_i$, $\Pr (x^{(i)}_j \text{ is } \text{ large}) \le \rho ^{2^i+1}$.

We start by considering the base case $i=0$. Each element $x^{(0)}_j \in S_i$ is obtained by running *FindMin* on a (multi)set $X_j$ of $\big \lceil 2\log \frac{1}{\rho } \big \rceil$ elements sampled, with replacement, from *S*. In order for $x^{(0)}_j$ to be large, at least one of the following conditions must be true: (i) the execution of *FindMin* on $X_j$ fails, which happens with probability at most $\varrho =\frac{\rho ^2}{2}$, or (ii) all elements in $X_j$ are large. Since each element in $X_j$ is independently large with probability at most $\frac{1}{4}$, the probability of (ii) is at most $\frac{1}{4^{2\log \frac{1}{\rho }}} = \rho ^4$. Using the union bound and $\rho \le \frac{1}{2}$, we have that $x^{(0)}_j$ is large with probability at most $\rho ^4 + \frac{\rho ^2}{2} \le \frac{\rho ^2}{4} + \frac{\rho ^2}{2} < \rho ^2 = \rho ^{2^i+1}$.

We now consider $i \ge 1$ and we show that if the induction claim holds for $i-1$ then it must also hold for *i*. Since $i \ge 1$, we know that each element $x^{(i)}_j \in S_i$ is the winner of a match between the elements $x^{(i-1)}_{2j-1}$ and $x^{(i-1)}_{2j}$ in $S_{i-1}$, each of which is large with probability at most $\rho ^{2^{i-1}+1}$ by the induction hypothesis. Moreover, each $x^{(i-1)}_j$ is chosen as function of a collection $C(i-1,j) = \{ x^{(0)}_h \mid j 2^{i-1} \le h < (j+1) 2^{i-1} \}$ of elements from $S_0$ along with all the outcomes of their pairwise comparisons performed during phases $1, \dots , i-1$. Since $C(i-1, 2j-1)$ and $C(i-1, 2j)$ are disjoint subsets of $S_0$, and the elements $x^{(0)}_h$ in $S_0$ are chosen using independent executions of *FindMin* (on independently chosen subsets $X_h$ of *S*), we have that the events “$x^{(i-1)}_{2j-1}$ is large” and “$x^{(i-1)}_{2j}$ is large” are also independent. For $x^{(i)}_j$ to be large either (i) $x^{(i-1)}_{2j-1}$ and $x^{(i-1)}_{2j}$ are both large, which happens with probability at most $(\rho ^{2^{i-1} + 1})^2 = \rho ^{2^i+2}$, or (ii) exactly one of $x^{(i-1)}_{2j-1}$ and $x^{(i-1)}_{2j}$ is large and it wins the match in phase *i*. The probability that exactly one of $x^{(i-1)}_{2j-1}$ and $x^{(i-1)}_{2j}$ is large can be upper bounded by the probability that at least one of $x^{(i-1)}_{2j-1}$ and $x^{(i-1)}_{2j}$ is large, which is at most $2 \cdot \rho ^{2^{i-1} + 1} \ge 2 \rho ^2$ by induction hypothesis. We hence focus on the probability that, in a match between a large and a small element, the large element wins. Since $x^{(i-1)}_{2j-1}$ and $x^{(i-1)}_{2j}$ are compared $2 c_p \big \lceil 2^i \ln \frac{1}{\rho } \big \rceil + 3$ times during the match, Lemma 1 ensures that this probability is at most $e^{ - 2^i \ln \frac{1}{\rho } - 1 } = \rho ^{2^i + 1}$. Putting it all together, we have:

$$\begin{aligned}&\Pr \big (x^{(i)}_j \text{ is } \text{ large }\big ) \le \rho ^{2^i+2} + 2\rho ^2 \cdot \rho ^{2^i + 1} \\&\quad = (\rho + 2\rho ^2) \rho ^{2^i + 1} \le \left( \frac{1}{2} + \frac{2}{4}\right) \rho ^{2^i + 1} = \rho ^{2^i + 1}. \end{aligned}$$

This completes the proof by induction and shows that the winner of the tournament (i.e., the sole element in $S_{\log n}$) is large with probability at most $\rho ^{2^{\log n} +1 } = \rho ^{n+1}$ (and small with probability at least $1- \rho ^{n+1}$). $\square$

We now analyze the number of comparisons performed by our algorithm.

### Lemma 7

Simulating the tournament requires $O(n \cdot \log n \cdot \log \frac{1}{\rho } + n \cdot \log ^2 \frac{1}{\rho })$ comparisons in the worst-case.

### Proof

The initial selection of the elements in $S_0$ requires $O(n \cdot \log \frac{1}{\rho } \cdot \log \frac{1}{\varrho }) = O(n \cdot \log ^2 \frac{1}{\rho })$ comparisons (recall that we choose $\varrho = \frac{\rho ^2}{2}$). The tournament itself consists of $\log n$ rounds. The number of matches that take place in round *i* is $\frac{n}{2^i}$ and, for each match, $O( 2^i \log \frac{1}{\rho } )$ comparisons are needed. It follows that the total number of comparisons performed in each round is $O(n \log \frac{1}{\rho })$ and, since there are $\log n$ rounds, the overall number of comparisons in rounds 1 to $\log n$ is $O(n \cdot \log n \cdot \log \frac{1}{\rho })$. $\square$

If we now select $\rho = \min \big \{ \frac{1}{2}, q^{\frac{1}{n}} \big \}$, we obtain an algorithm for ${{\textsc {FT}}\hbox {-}{\textsc {Min}}} \big (\frac{3}{4}n \big )$ that performs $O\Big ( n \log n + \log n \cdot \log \frac{1}{q} + \frac{\log ^2 \frac{1}{q}}{n}\Big )$ comparisons in the worst case and has a success probability of at least $1- \rho ^{n+1} \ge 1- \rho \cdot (q^{\frac{1}{n}})^n = 1- \rho \cdot q \ge 1 - \frac{q}{2}$. We can now use this algorithm in our reduction of Lemma 3 to immediately obtain an algorithm for ${{\textsc {FT}}\hbox {-}{\textsc {Min}}} (k)$ which is optimal for $k = O(\frac{n}{\log \log \frac{1}{q}})$ and *q* bounded away from $q_{\mathrm{crit}}$ (see Theorem 5 in Sect. 6).

### Theorem 3

${{\textsc {FT}}\hbox {-}{\textsc {Min}}} (k)$ can be solved with a success probability of at least $1-q$ using $O\big (\frac{n}{k} \log \frac{1}{q} + (\log \frac{1}{q}) \log \log \frac{1}{q} \big )$ comparisons in the worst case.

We can combine this algorithm with *GeometricTest* (described in Sect. 4) to solve ${{\textsc {FT}}\hbox {-}{\textsc {Min}}} (k)$ with a success probability of at least $1-\frac{1}{q}$ using *both* $O(\frac{n}{k} \log \frac{1}{q})$ comparisons in expectation and $O\big (\frac{n}{k} \log \frac{1}{q} + (\log \frac{1}{q}) \log \log \frac{1}{q}\big )$ comparisons in the worst case. In order to do so, we simply run the two algorithms in parallel until one of them terminates. Clearly, the expected number of comparisons is asymptotically unaffected, while the probability that this combined algorithm fails can be upper bounded by the sum of the respective failure probabilities, i.e., by at most $\frac{q}{4} + \frac{q}{2} < q$ (recall that *GeometricTest* fails with probability at most $\frac{q}{4}$, as shown by Lemma 5).

## Lower Bound

**Fig. 2:**
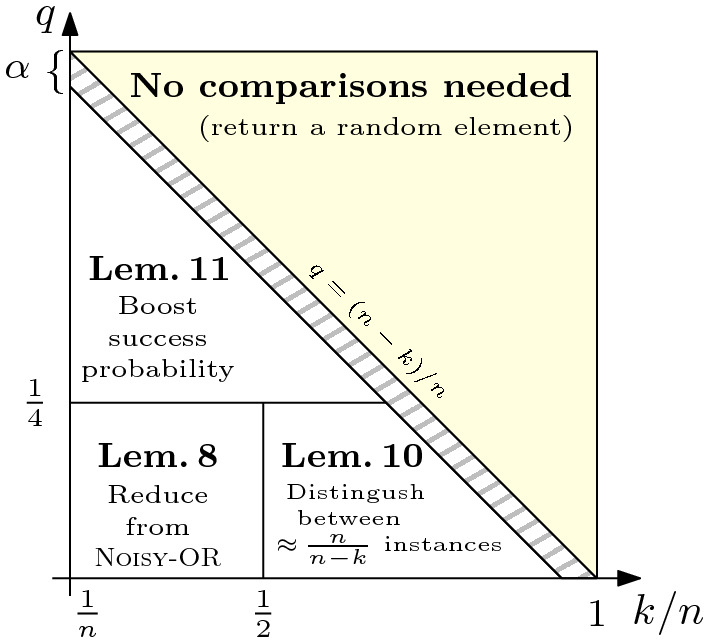
A qualitative representation of the different ranges of the parameters *q* and *k* (as a fraction of *n*) handled by Lemmas 8, 10, and 11. The figure is not to scale

The rest of the paper is devoted to proving our lower bound of ${\varOmega }(\frac{n}{k} \log \frac{1}{q})$ on the *expected* number of comparisons required to solve ${{\textsc {FT}}\hbox {-}{\textsc {Min}}} (k)$ with a success probability of at least $1-q$. We prove our lower bound using three different strategies depending on the values of *k* and *q*. Figure 2 shows a qualitative representation of the considered regions.

We start by considering what we deem to be the most interesting case, namely the one in which $n \ge 2k$ and *q* can be upper bounded by a small enough constant. For simplicity we pick this constant to be $\frac{1}{4}$, although the same proof strategy actually works for any $q \le \frac{1}{2}-\varepsilon$, where $\varepsilon >0$ is a constant of choice.^9^ We will show that any algorithm that is able to solve ${{\textsc {FT}}\hbox {-}{\textsc {Min}}} (k)$ with a success probability of at least $1-q$ can also be used to solve ${{\textsc {FT}}\hbox {-}{\textsc {Min}}} (1)$ with the same success probability. Then, our lower bound for ${{\textsc {FT}}\hbox {-}{\textsc {Min}}} (k)$ follows from the fact that any algorothm that solves ${{\textsc {FT}}\hbox {-}{\textsc {Min}}} (1)$ with a success probability of a least $1-q \in \left[ \frac{3}{4}, 1\right)$ must perform ${\varOmega }(\log \frac{1}{q})$ comparison in expectation. This is formalized in the following theorem, whose proof is given in Appendix A and is similar to the one used in [14, Theorem 2.1] to establish a lower bound on the *worst-case* number of comparisons needed to solve ${{\textsc {FT}}\hbox {-}{\textsc {Min}}} (1)$.^10^

### Theorem 4

Let *A* be an algorithm that solves ${{\textsc {FT}}\hbox {-}{\textsc {Min}}} (1)$ with a probability of success of at least $1-q \in (\frac{1}{2}, 1]$. For any $n \ge 2$, there exists a sequence *S* of *n* elements such that the expected number of comparisons performed by *A* on *S* is at least $\gamma n \log \frac{1}{2q}$, where $\gamma >0$ is a constant that depends only on *p*.

We are now ready to prove our lower bound for ${{\textsc {FT}}\hbox {-}{\textsc {Min}}} (k)$ when $n \ge 2k$ and $q \le \frac{1}{4}$.

### Lemma 8

Let $n \ge 2k$ and $q \le \frac{1}{4}$. For every algorithm *A* that solves ${{\textsc {FT}}\hbox {-}{\textsc {Min}}} (k)$ with a success probability of at least $1-q$, there exists a sequence *S* of *n* elements such that the expected number of comparisons performed by *A* on *S* is larger than $\psi _1 \frac{n}{k} \log \frac{1}{q}$, where $\psi _1 >0$ is a constant that depends only on *p*.

### Proof

Let $\gamma$ be the constant from Theorem 4, choose $\psi _1 = \frac{\gamma }{4}$, and suppose towards a contradiction that there is an algorithm *A* that is able to solve ${{\textsc {FT}}\hbox {-}{\textsc {Min}}} (k)$ with a success probability of at least $1-q$ using an expected number of comparisons of at most $\psi _1 \frac{n}{k} \log \frac{1}{q}$ on every instance of $n \ge 2k$ elements. We will show that the existence of *A* implies the existence of an algorithm $A'$ that is able to solve ${{\textsc {FT}}\hbox {-}{\textsc {Min}}} (1)$ with a success probability of at least $1-q$ on any instance of $n' = \left\lfloor \frac{n}{k}\right\rfloor \ge 2$ elements using fewer than $\gamma n' \log \frac{1}{2q}$ comparisons in expectation, thus contradicting Theorem 4.

Algorithm $A'$ works as follows: given an instance $S'$ of ${{\textsc {FT}}\hbox {-}{\textsc {Min}}} (1)$ with $n'$ elements, $A'$ constructs an instance *S* of ${{\textsc {FT}}\hbox {-}{\textsc {Min}}} (k)$ that consists of *k* copies of each element in $S'$ and of $n - k \left\lfloor \frac{n}{k}\right\rfloor < k$ copies of an arbitrary element from $S'$. Then, $A'$ runs *A* on *S* (which contains exactly *n* elements) and outputs the element *x* returned by the execution of *A*. With probability at least $1-q$, *x* is among the *k* smallest elements of *S*, implying that it is (a copy of) the smallest element of $S'$.

To conclude the proof, it suffices to notice that the expected number of comparisons performed by $A'$ on $S'$ is upper bounded by the expected number of comparisons performed by *A* on *S*, i.e., it is at most

$$\begin{aligned} \psi _1 \frac{n}{k} \log \frac{1}{q} = \frac{\gamma }{2} \frac{n}{2k} \log \frac{1}{q} \le \gamma \frac{n}{2k} \log \frac{1}{2q} \le \gamma \left( \frac{n}{k} - 1\right) \log \frac{1}{2q} < \gamma n' \log \frac{1}{2q}. \end{aligned}$$

$$\square $$

We now turn our attention to the ranges of *k* and *q* that are not covered by Lemma 8. Recall that for $q \ge q_{\mathrm{crit}}= \frac{n-k}{n}$, no lower bound exists since ${{\textsc {FT}}\hbox {-}{\textsc {Min}}} (k)$ can be solved *without performing any comparison* by simply returning an element chosen uniformly at random from *S*. In the rest of this section we will consider values of *q* that are *bounded away* from $q_{\mathrm{crit}}$, namely we assume the existence of some *constant* $\alpha >0$ for which $0 < q \le \frac{n-k}{n} - \alpha$. As a consequence, we can prove a preliminary lower bound of $\alpha$ on the expected number of comparisons needed to solve ${{\textsc {FT}}\hbox {-}{\textsc {Min}}} {k}$ with a success probability of at least $1-q$. This lower bound will be useful to handle some corner cases in the sequel.

### Lemma 9

Let $n > k$ and $q \le \frac{n-k}{n} - \alpha$. For every algorithm *A* that solves ${{\textsc {FT}}\hbox {-}{\textsc {Min}}} (k)$ with a success probability of at least $1-q$, there exists a sequence *S* of *n* elements such that the expected number of comparisons performed by *A* on *S* is at least $\alpha$.

### Proof

Suppose towards a contradiction that there is an algorithm *A* that is able to solve ${{\textsc {FT}}\hbox {-}{\textsc {Min}}} (k)$ with a success probability of at least $1-q$ using an expected number of comparisons smaller than $\alpha$ on every instance of $n > k$ elements. Then, an execution of *A* performs no comparisons with a probability larger than $1-\alpha$. When the input of *A* is a random permutation of $\langle 1, 2, \dots , n \rangle$, the failure probability must be larger than $(1-\alpha ) \frac{n-k}{n} = \frac{n-k}{n} - \frac{\alpha (n-k)}{n} > \frac{n-k}{n} - \alpha \ge q$. This implies the existence of at least one instance of *n* elements for which *A* fails with a probability larger than *q*, yielding the sought contradiction. $\square$

We now handle the case $n < 2k$ and $q \le \frac{1}{4}$. We will consider a suitable set of instances ensuring that any algorithm having a success probability of at least $1-q$ must perform at least $\psi _2 \log \frac{1}{q}$ comparisons on at least one instance in the set, for some constant $\psi _2 > 0$. Notice that, in this case, $\psi _2 \log \frac{1}{q} > \frac{\psi _2}{2} \frac{n}{k}\log \frac{1}{q}$.

### Lemma 10

Let $k< n < 2k$ and $q \le \min \{\frac{1}{4}, \frac{n-k}{n} - \alpha \}$. For every algorithm *A* that solves ${{\textsc {FT}}\hbox {-}{\textsc {Min}}} (k)$ with a success probability of at least $1-q$, there exists a sequence *S* of *n* elements such that the expected number of comparisons performed by *A* on *S* is at least $\psi _2 \log \frac{1}{q}$, where $\psi _2 >0$ is a constant that depends only on $\alpha$ and *p*.

### Proof

Let *A* be an algorithm that solves ${{\textsc {FT}}\hbox {-}{\textsc {Min}}} (k)$ with a success probability of at least $1-q \ge \frac{3}{4}$ using at most $\mu$ comparisons in expectation on every instance of *n* elements, where $k< n < 2k$.

Let $\beta = \frac{3 \alpha }{8(\alpha +1)}$, notice that $\beta \in (0,1)$ is a constant as it only depends on $\alpha$, and define $\psi _2 = \min \left\{ \frac{\alpha }{2\log \frac{1}{\beta }}, \frac{ 1 }{8\log \frac{1-p}{p}} \right\}$. We only need to consider $q < \beta ^2$ since, when $q \ge \beta ^2$, Lemma 9 already ensures that $\mu \ge \alpha \ge \frac{\alpha }{2\log \frac{1}{\beta }} \log \frac{1}{q} \ge \psi _2 \log \frac{1}{q}$.

In the rest of the proof we will consider $\eta +1$ sequences ${\mathcal {I}}_0, {\mathcal {I}}_1, \dots {\mathcal {I}}_\eta$ having *n* elements each, where $\eta = \left\lceil \frac{k}{n-k} \right\rceil$. Then, we will lower bound $\mu$ by considering the expected number of comparisons needed by *A* to solve each instance ${\mathcal {I}}_i$ with a success probability of at least $1-q$.

**Fig. 3:**
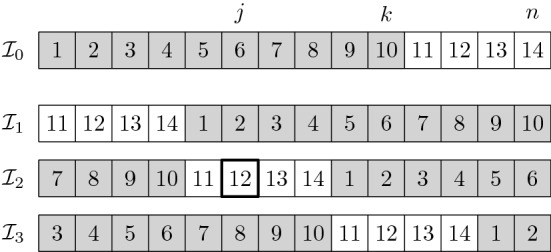
An example of the input sequences ${\mathcal {I}}_0, \dots , {\mathcal {I}}_\eta$ used in the proof of Lemma 10 for $n=14$ and $k=10$ (in this case $\eta =3$). The positions in $L_1, \dots , L_\eta$ have a white background, while those containing the *k* smallest elements of each sequence have a gray background (since $k>\frac{n}{2}$, the gray intervals of any two sequences must overlap). Each position $j \in \mathcal \{1, \dots , k\}$ contains one of the smallest *k* elements in at least one of the sequences. In particular, our choice of $j^*$ ensures that $x^{(j^*)}_j$ is not among the *k* smallest elements of ${\mathcal {I}}_{j^*}$, i.e., $j \in L_{j^*}$. The element $x^{(j^*)}_j$ for $j=6$ is highlighted in bold (in this case $j^*= \lceil 6/(14-10) \rceil = 2$)

We start by defining ${\mathcal {I}}_0 = \langle 1, 2, \dots , n \rangle$ and, for $i=1,\dots ,\eta$, we let ${\mathcal {I}}_i$ be the sequence obtained by performing $(n-k)\cdot i$ consecutive right rotations on ${\mathcal {I}}_0$.^11^ See Fig. 3 for an example. We denote by $x^{(i)}_j$ the element that appears in the *j*-th position of sequence ${\mathcal {I}}_i$. We will use $A({\mathcal {I}}_i)$ to refer to an execution of *A* with input ${\mathcal {I}}_i$, and $A({\mathcal {I}}_i)=x^{(i)}_j$ to denote the event “$A({\mathcal {I}}_i)$ returns element $x^{(i)}_j$”. Moreover, we let $F_i$ be the event “$A({\mathcal {I}}_i)$ performs at most $4\mu$ comparisons”. Since the expected number of comparisons of $A(I_i)$ is at most $\mu$, the Markov inequality implies that $A(I_i)$ performs more than $4\mu$ comparisons with a probability of at most $\frac{1}{4}$, i.e., $\Pr (F_i) \ge \frac{3}{4}$.

Given any input sequence of *n* elements and $j \in \{1, \dots , n\}$, we can encode an execution of *A* that returns the *j*-th element of the input sequence with a pair (*C*, *R*) where *C* is the list of the observed comparison outcomes, and *R* is the list of all random choices taken by *A*. Let $\Pr \big ( (C,R) \mid {\mathcal {I}}_i \big )$ be the probability that the execution (*C*, *R*) of *A* is realized when when the input sequence is ${\mathcal {I}}_i$. Moreover, let $\Pr (R \mid C)$ denote the probability that the random choices of an execution of *A* are exactly those in *R*, given that the observed comparison results match those in *C*. Consider the case in which each comparison error is exactly *p*. Then, the probability of observing the comparison outcomes in *C* when *A*’s input is ${\mathcal {I}}_0$ (resp. ${\mathcal {I}}_i$) is *at most* $(1-p)^{|C|}$ (resp. *at least* $p^{|C|}$), allowing us to relate $\Pr \big ( (C,R) \mid {\mathcal {I}}_0 \big )$ and $\Pr \big ( (C,R) \mid {\mathcal {I}}_i \big )$ as follows:

$$\begin{aligned} \Pr \big ( (C,R) \mid {\mathcal {I}}_0 \big )&\le (1-p)^{|C|} \Pr (R \mid C) = \left( \frac{1-p}{p}\right) ^{|C|} p^{|C|} \Pr (R \mid C) \\&\le \left( \frac{1-p}{p}\right) ^{|C|} \Pr \big ( (C,R) \mid {\mathcal {I}}_i \big ). \end{aligned}$$

By summing the above inequality over all the choices of (*C*, *R*) for which *A* returns the *j*-th input element and such that $|C| \le 4\mu$, we obtain $\Pr \left( A({\mathcal {I}}_0) = x^{(0)}_j \mid F_0 \right) \le \left( \frac{1-p}{p}\right) ^{4\mu } \Pr \left( A({\mathcal {I}}_i) = x^{(i)}_j \mid F_i\right)$. Let $L_i$ be the set of all indices $h \in \{1, \dots , n\}$ such that $x^{(i)}_h$ is not among the *k* smallest elements of ${\mathcal {I}}_i$. Moreover, for $j \in \{1, \dots , k\}$, define $j^* = \left\lceil \frac{j}{n-k} \right\rceil$ and notice that $j \in L_{j^*}$ (see Fig. 3). Then:

1 $$\begin{aligned} \Pr \left( A({\mathcal {I}}_0) \text{ suceeds } \mid F_0\right)&= \sum _{j=1}^k \Pr \left( A({\mathcal {I}}_0) = x_j^{(0)} \mid F_0\right) \nonumber \\&\le \left( \frac{1-p}{p}\right) ^{4\mu } \sum _{j=1}^k \Pr \left( A({\mathcal {I}}_{j^*}) = x_j^{(j^*)} \mid F_{j^*}\right) \end{aligned}$$

2 $$\begin{aligned}&\le \left( \frac{1-p}{p}\right) ^{4\mu } \sum _{i=1}^{\eta } \sum _{h \in L_i} \Pr \left( A({\mathcal {I}}_{i}\right) = x_h^{(i)} \mid F_i) \nonumber \\&= \left( \frac{1-p}{p}\right) ^{4\mu } \sum _{i=1}^{\eta } \Pr \left( A({\mathcal {I}}_i) \text{ fails } \mid F_i\right) , \end{aligned}$$

where the second inequality follows from the fact that the generic *j*-th summand in (1) appears in the double sum of (2) when $i=j^*$ and $h=j \in L_{j^*}$. Since we know that, for all $i=0,\dots ,\eta$, $A({\mathcal {I}}_i)$ fails with probability at most $q \le \frac{1}{4}$, we have:

$$\begin{aligned} \eta q&\ge \sum _{i=1}^\eta \Pr (A({\mathcal {I}}_i) \text{ fails } ) \ge \sum _{i=1}^\eta \Pr (A({\mathcal {I}}_i) \text{ fails } \cap F_i) =\sum _{i=1}^\eta \Pr (A( {\mathcal {I}}_i) \text{ fails } \mid F_i) \Pr (F_i) \\&\ge \frac{3}{4} \sum _{i=1}^\eta \Pr (A({\mathcal {I}}_i) \text{ fails } \mid F_i) \ge \frac{3}{4} \left( \frac{p}{1-p} \right) ^{4\mu } \Pr (A({\mathcal {I}}_0) \text{ suceeds } \mid F_0) \\&\ge \frac{3}{4} \left( \frac{p}{1-p} \right) ^{4\mu } \Pr (A({\mathcal {I}}_0) \text{ suceeds } \cap F_0) \ge \frac{3}{8} \left( \frac{p}{1-p} \right) ^{4\mu }, \end{aligned}$$

The above inequality yields $\mu \ge \frac{ \log \frac{3}{8 \eta q} }{4\log \frac{1-p}{p}}$, which can be combined with $\eta \le 1+ \frac{n}{n-k} \le 1+ \frac{1}{q+\alpha } \le 1 + \frac{1}{\alpha } = \frac{\alpha +1}{\alpha }$ to obtain the sought lower bound on $\mu$:

$$\begin{aligned} \mu&\ge \frac{ \log \frac{3}{8 \eta q} }{4\log \frac{1-p}{p}} \ge \frac{ \log \frac{3 \alpha }{8 (\alpha +1) q} }{4\log \frac{1-p}{p}} = \frac{ \log \frac{\beta }{q} }{4\log \frac{1-p}{p}} = \frac{ \frac{1}{2}\log \beta ^2 + \log \frac{1}{q} }{4\log \frac{1-p}{p}} \\&> \frac{ \frac{1}{2}\log q + \log \frac{1}{q} }{4\log \frac{1-p}{p}} = \frac{ \log \frac{1}{q} }{8\log \frac{1-p}{p}} \ge \psi _2 \log \frac{1}{q}. \end{aligned}$$

$$\square $$

Finally, we consider the remaining case $q > \frac{1}{4}$. We show that the success probability of any algorithm that solves ${{\textsc {FT}}\hbox {-}{\textsc {Min}}} (k)$ can be boosted from $1-q$ to at least $\frac{1}{4}$ by running it multiple times and selecting the smallest returned element using ${\textit{FindMin}}$. Then, the lower bound of either Lemmas 8 or 10 applies.

### Lemma 11

Let $n > k$ and $\frac{1}{4} < q \le \frac{n-k}{n} - \alpha$. For every algorithm *A* that solves ${{\textsc {FT}}\hbox {-}{\textsc {Min}}} (k)$ with a probability of success of at least $1-q$, there exists a sequence *S* of *n* elements such that the expected number of comparisons performed by *A* on *S* is at least $\psi _3 \frac{n}{k} \log \frac{1}{q}$, where $\psi _3 >0$ is a constant that depends only on $\alpha$ and *p*.

### Proof

Let $c \ge 2$ be a constant such that ${\textit{FindMin}}$ with a failure probability of $\varrho =\frac{1}{8}$ requires at most *cm* comparisons on any instance of *m* elements (Theorem 1 ensures that such a constant exists). Let *A* be an algorithm that solves ${{\textsc {FT}}\hbox {-}{\textsc {Min}}} (k)$ with a probability of error of at most *q* using an expected number of comparisons of at most $\mu$ on every instance of *n* elements.

Let $\psi _1$ and $\psi _2$ be the constants of Lemmas 8 and 10, respectively. Define $\psi ' = \min \{\psi _1, \frac{\psi _2}{2}\}$, $\beta = \frac{10}{\psi '} \log ^{-2} \frac{1}{1-\alpha }$, and $\psi _3 = \min \left\{ \frac{\alpha }{2 \beta c}, \frac{\psi '}{10} \log \frac{1}{1-\alpha } \right\}$. We can restrict ourselves to the case $\frac{n}{k} > \beta c$ since otherwise we can use Lemma 9 and the inequality $\log \frac{1}{q} \le 2$ to write $\mu \ge \alpha \ge \frac{\alpha }{\beta c} \frac{n}{k} \ge \frac{\alpha }{2 \beta c} \frac{n}{k} \log \frac{1}{q} \ge \psi _3 \frac{n}{k} \log \frac{1}{q}$.

We now describe an algorithm $A'$ that uses *A* to solve ${{\textsc {FT}}\hbox {-}{\textsc {Min}}} (k)$ with a success probability of at least $\frac{3}{4}$. Given an instance *S* of *n* elements, $A'$ works as follows: first, it performs $t= \left\lceil \frac{3}{\log \frac{1}{q}} \right\rceil$ independent executions of *A* with input *S* and collects the returned elements into a set $S'$; then, it runs *FindMin* on $S'$ with a probability of error of $\varrho =\frac{1}{8}$, and answer with the returned element.

The expected number of comparisons of $A'$ is at most $ct + t \mu$, and the probability that no execution of *A* returns one of the *k* smallest elements is at most $q^t \le q^{\frac{3}{\log \frac{1}{q}}} = \left( \frac{1}{2} \right) ^3 = \frac{1}{8}$. By the union bound, the overall failure probability of $A'$ is at most $\frac{1}{8} + \frac{1}{8} = \frac{1}{4}$. By invoking either Lemmas 8 or 10 (depending on the values of *k* and *n*), we know that the expected number of comparisons of $A'$ must be at least $\min \{ \psi _1, \frac{\psi _2}{2}\} \frac{n}{k} \log \frac{1}{q} = \psi ' \frac{n}{k} \log \frac{1}{q}$. In formulas:

3 $$\begin{aligned} tc + t\mu \ge \psi ' \frac{n}{k} \log \frac{1}{q}. \end{aligned}$$

Since $\frac{3}{\log \frac{1}{q}} > \frac{3}{2}$, we have $t = \left\lceil \frac{3}{\log \frac{1}{q}} \right\rceil \le \frac{3}{\log \frac{1}{q}} + 1 < (1+ \frac{2}{3}) \frac{3}{\log \frac{1}{q}} = \frac{5}{\log \frac{1}{q}}$. Moreover, we know that $q \le \frac{n-k}{n} - \alpha < 1-\alpha$. We can then solve (3) for $\mu$ and combine the result with the above inequalities:

4 $$\begin{aligned} \mu \ge \frac{ \psi ' n}{tk} \log \frac{1}{q} -c> \frac{\psi ' n}{5 k} \log ^2 \frac{1}{q} - c > \left( \frac{\psi '}{5} \log \frac{1}{1-\alpha } \right) \cdot \frac{n}{k} \log \frac{1}{q} - c. \end{aligned}$$

Using $q < 1-\alpha$ once more, together with $\frac{n}{k} > \beta c$, we have:

$$\begin{aligned} \left( \frac{\psi '}{5} \log \frac{1}{1-\alpha } \right) \cdot \frac{n}{k} \log \frac{1}{q}> \frac{\psi ' n}{5 k} \log ^2 \frac{1}{1-\alpha } = 2 \beta ^{-1} \frac{n}{k} > 2c, \end{aligned}$$

which can be combined with (4) to show that $\mu > \left( \frac{\psi '}{10} \log \frac{1}{1-\alpha } \right) \cdot \frac{n}{k} \log \frac{1}{q} \ge \psi _3 \frac{n}{k} \log \frac{1}{q}$. $\square$

Combining Lemmas 8, 10, and 11, we obtain the main result of this section:

### Theorem 5

Let $n > k$ and $q \le \frac{n-k}{n} - \alpha$. For every algorithm *A* that solves ${{\textsc {FT}}\hbox {-}{\textsc {Min}}} (k)$ with a success probability of at least $1-q$, there exists a sequence *S* of *n* elements such that the expected number of comparisons performed by *A* on *S* is at least $\psi \frac{n}{k} \log \frac{1}{q}$, where $\psi >0$ is a constant that depends only on $\alpha$ and *p*.

## Proof of Theorem 4

Consider the following Noisy-OR problem, first introduced in [14]: we are given a finite input sequence $\langle y_1, y_2, \dots \rangle$ where $y_i \in \{0,1\}$ and we need to output “true” if at least one $y_i$ equals 1, and “false” otherwise. The actual value of an element $y_i$ cannot be read directly but we can perform queries to a noisy oracle ${\mathcal {O}}$. Whenever ${\mathcal {O}}$ is queried with an element $y_i$, it returns $y_i$ with probability $1-p$ and $1-y_i$ with the complementary probability. We will denote such a query by ${\mathcal {O}}(y_i)$.

Given an algorithm *A* that solves ${{\textsc {FT}}\hbox {-}{\textsc {Min}}} (1)$ on every instance of *n* elements with a success probability of at least $1-q$, we can design an algorithm $A'$ that solves the Noisy-OR problem on every instance of $n-1$ elements with the same success probability. Intuitively, algorithm $A'$ on input $\langle y_1, y_2, \dots , y_{n-1} \rangle$ simulates an execution of *A* on input $\langle x_1, x_2, \dots , x_{n-1}, x_n \rangle$ where $x_n = n$ and, for $i=1, \dots , n-1$, $x_i = (1 - y_i)n + i$. As a consequence, with probability at least $1-q$, $A'$ returns $x_n$ if all $y_i$s are 0, and $x_i \ne x_n$ where *i* is the smallest index such that $y_i = 1$ otherwise. Formally $A'$ executes *A* with the following modifications:

- Whenever *A* compares two elements $x_i$ and $x_j$ with $i<j$ (the case $j>i$ is symmetric) $A'$ simulates the comparison as follows:
  - If $j=n$, then $A'$ queries ${\mathcal {O}}$ with $y_i$. If ${\mathcal {O}}(y_i)$ returns “1”, $x_i$ is treated as smaller than $x_n$. Otherwise, $x_n$ is treated as smaller than $x_i$.
  - If $j \ne n$, then $A'$ performs $2 c_p \big \lceil \ln \frac{2}{p} \big \rceil + 1$ queries ${\mathcal {O}}(y_i)$ (resp. ${\mathcal {O}}(y_j)$), and computes their majority result $r_i$ (resp $r_j$). If exactly one $r_h \in \{r_i, r_j\}$ is 1, $A'$ treats the corresponding element $x_h$ as the smaller between $x_i$ and $x_j$. Otherwise (if $r_i = r_j$) $x_i$ is treated as smaller than $x_j$. Notice that, by Lemma 1, $r_i = x_i$ (resp. $r_j = x_j$) with probability at least $1-\frac{p}{2}$, showing that the element that is reported as smaller is actually the smaller between $x_i$ and $x_j$ with a probability of at least $1 - 2 \frac{p}{2} = 1-p$.
- Whenever *A* terminates and returns an element $x_i$, $A'$ returns “true” if $x_i \ne x_n$ and “false” otherwise.

Each comparison of *A* is implemented by $A'$ using at most $4 c_p \big \lceil \ln \frac{2}{p} \big \rceil + 2$ queries to ${\mathcal {O}}$, therefore if the average number of comparisons required *A* on any instance of *n* elements is at most $\mu$, $A'$ performs at most $(4 c_p \big \lceil \ln \frac{2}{p} \big \rceil + 2) \mu$ queries in expectation on every instance of Noisy-OR consisting of $n-1$ elements. In the rest of the proof we will show that no algorithm for Noisy-OR having a success probability of at least $1-q$ can perform fewer than $\gamma ' n \log \frac{1}{2q}$ queries on all instances of $n-1$ elements, where $\gamma '>0$ is a suitable constant that depends on *p*. This will immediately prove that $\mu \ge \frac{\gamma '}{4 c_p \lceil \ln \frac{2}{p} \rceil + 2} n \log \frac{1}{2q} = \gamma n \log \frac{1}{2q}$, where $\gamma = \frac{\gamma '}{4 c_p \lceil \ln \frac{2}{p} \rceil + 2}$.

Consider any algorithm ${\overline{A}}$ that solves Noisy-OR with a success probability of at least $1-q$ and a let $\nu$ be the maximum among the expected number of queries performed by ${\overline{A}}$ on any input sequence of $n-1$ elements. We will derive a lower bound on $\nu$. Assume that $\nu$ is finite and let $\varepsilon \in (0, \frac{1}{2q}-1)$ be a parameter that will be chosen later. The existence of ${\overline{A}}$ implies the existence of an algorithm $A^*$ for Noisy-OR that has a success probability of at least $1-(1+\varepsilon )q$, performs at most $\nu$ queries in expectation (on every instance of $n-1$ elements), and at most $\big \lceil \frac{\nu }{q\varepsilon } \big \rceil$ queries in the worst case. Such $A^*$ can be obtained from ${\overline{A}}$ by stopping ${\overline{A}}$ immediately before the $\left( \big \lceil \frac{\nu }{q\varepsilon } \big \rceil +1\right)$-th query to ${\mathcal {O}}$ (if ${\overline{A}}$ is stopped in this way, $A^*$ returns any arbitrary input element). By the Markov inequality ${\overline{A}}$ is stopped with probability at most $\frac{\nu }{\nu / (q\varepsilon )} = q\varepsilon$, showing that the probability of failure $q'$ of $A^*$ is at most $q + q\varepsilon = (1+\varepsilon )q < \frac{1}{2}$.

For $i=0,\dots , n-1$, let ${\mathcal {I}}_i$ denote the sequence $\langle y_1, \dots , y_{n-1} \rangle$, where $y_j = 1$ if $j=i$ and $y_j = 0$ otherwise (so that ${\mathcal {I}}_0$ consists of $n-1$ zeros). Similarly to the proof of Theorem 2.1 in [14], we consider the rooted *noisy Boolean decision tree* *T* associated with $A^*$: each internal vertex *v* of *T* has a label $\lambda _v \in \{1, \dots , n-1\}$ and represents a query ${\mathcal {O}}(y_{\lambda _v})$, while each leaf is labeled with either “true” or “false”. A specific execution of $A^*$ on an input sequence of $n-1$ elements traces a path $P_\ell$ between the root *r* of *T* and a leaf $\ell$ of *T*, and the output of the execution matches the label of $\ell$. Let *L* be the set of leaves of *T*.

Given $\ell \in L$ we will denote by $\Pr (\ell \mid {\mathcal {I}}_i)$ the probability that an execution of $A^*$ on input ${\mathcal {I}}_i$ traces the (unique) path $P_\ell$ from *r* to $\ell$ in *T*. For $j=1,\dots ,n-1$, define $\eta (\ell , j)$ as the number of internal vertices labeled *j* on the unique path $P_\ell$ between the root of *T* and $\ell$. It follows that the execution of $A^*$ described by $P_\ell$ performs $\sum _{j=1}^{n-1} \eta (\ell , j) = d(\ell )$ queries to ${\mathcal {O}}$, where $d(\ell )$ is the depth of $\ell$ in *T*.

For $i=1,\dots ,n-1$, the only input element that differs between ${\mathcal {I}}_i$ and ${\mathcal {I}}_0$ is $y_i$. Since an execution of $A^*$ that traces $P_\ell$ performs $\eta (\ell , i)$ queries ${\mathcal {O}}(y_i)$, we have that $\Pr (\ell \mid {\mathcal {I}}_i)$ must be at least $\left( \frac{p}{1-p} \right) ^{\eta (\ell , i)} \Pr (\ell \mid {\mathcal {I}}_0)$. Then:

5 $$\begin{aligned}&\sum _{i=1}^{n-1} \Pr (\ell \mid {\mathcal {I}}_i) \nonumber \\&\quad \ge \sum _{i=1}^{n-1} \left( \frac{p}{1-p} \right) ^{\eta (\ell , i)} \! \Pr (\ell \mid {\mathcal {I}}_0) = (n-1) \Pr (\ell \mid {\mathcal {I}}_0) \cdot \frac{1}{n-1} \sum _{i=1}^{n-1} \left( \frac{p}{1-p} \right) ^{\eta (\ell , i)} \!\!.\nonumber \\ \end{aligned}$$

Let $\phi (x) = \left( \frac{p}{1-p} \right) ^x$. Since $\phi (x)$ is a convex function we can use Jensen’s inequality to write:

6 $$\begin{aligned} \begin{aligned}&\frac{1}{n-1} \sum _{i=1}^{n-1} \left( \frac{p}{1-p} \right) ^{\eta (\ell , i)} = \frac{1}{n-1} \sum _{i=1}^{n-1} \phi (\eta (\ell , i)) \\&\ge \phi \left( \frac{1}{n-1} \sum _{i=1}^{n-1} \eta (\ell , i) \right) = \phi \left( \frac{d(\ell )}{n-1} \right) = \left( \frac{p}{1-p} \right) ^{\frac{d(\ell )}{n-1}}. \end{aligned} \end{aligned}$$

Let *Z* be the set of leaves of *T* labeled “false”. Combining (5) and (6), summing over all leaves $\ell \in Z$, and noticing that we must have $\sum _{\ell \in Z} \Pr (\ell \mid {\mathcal {I}}_i) \le q' \; \forall i = 1, \dots , n-1$, we obtain:

7 $$\begin{aligned} \begin{aligned} (n-1)q'&= \sum _{i=1}^{n-1} q' \ge \sum _{i=1}^{n-1} \sum _{\ell \in Z} \Pr (\ell \mid {\mathcal {I}}_i) = \sum _{\ell \in Z} \sum _{i=1}^{n-1} \Pr (\ell \mid {\mathcal {I}}_i) \\&\ge (n-1) \sum _{\ell \in Z} \left( \frac{p}{1-p} \right) ^{\frac{d(\ell )}{n-1}} \Pr (\ell \mid {\mathcal {I}}_0). \end{aligned} \end{aligned}$$

Define $\varphi (x) = \left( \frac{p}{1-p} \right) ^\frac{x}{n-1}$, $\alpha _\ell = \Pr (\ell \mid {\mathcal {I}}_0)$, and ${\overline{d}} = \sum _{\ell \in Z} \alpha _\ell d(\ell )$. Since $A^*$ succeeds with probability at least $1-q'$ on every input sequence of $n-1$ elements, we know that $\sum _{\ell \in Z} \alpha _\ell \ge 1-q'$. Moreover, $\phi (x)$ is a monotonically decreasing convex function, allowing us to combine the above inequality with Jensen’s inequality to write:

8 $$\begin{aligned} \begin{aligned}&\sum _{\ell \in Z} \left( \frac{p}{1-p} \right) ^{\frac{d(\ell )}{n-1}} \Pr (\ell \mid {\mathcal {I}}_0 ) = \sum _{\ell \in Z} \alpha _\ell \varphi (d(\ell )) \ge (1-q') \frac{ \sum _{\ell \in Z} \alpha _\ell \varphi (d(\ell )) }{\sum _{\ell \in Z} \alpha _\ell } \\&\quad \ge (1-q') \varphi \left( \frac{\sum _{\ell \in Z} \alpha _\ell d(\ell )}{\sum _{\ell \in Z} \alpha _\ell } \right) \\&\quad \ge (1-q') \varphi \left( \frac{{\overline{d}}}{1-q'} \right) = (1-q') \left( \frac{p}{1-p} \right) ^\frac{{\overline{d}}}{(1-q')(n-1)}. \end{aligned} \end{aligned}$$

From (7) and (8) we obtain:

$$\begin{aligned} (n-1)q' \ge (n-1) (1-q') \left( \frac{p}{1-p} \right) ^{\frac{{\overline{d}}}{(1-q')(n-1)}}, \end{aligned}$$

which implies

$$\begin{aligned} {\overline{d}} \ge \frac{(n-1) (1-q') \log \frac{q'}{1-q'}}{\log \frac{p}{1-p}} = \frac{(n-1) (1-q') \log \frac{1-q'}{q'}}{\log \frac{1-p}{p}}. \end{aligned}$$

We can lower bound $\nu$ with the average number ${\overline{d}}$ of queries performed by $A^*$ on input ${\mathcal {I}}_0$, indeed:

$$\begin{aligned} \nu \ge \sum _{\ell \in L} d(\ell ) \Pr (\ell \mid {\mathcal {I}}_0) \ge \sum _{\ell \in Z} d(\ell ) \Pr (\ell \mid {\mathcal {I}}_0) = {\overline{d}}. \end{aligned}$$

We now choose $\gamma ' = \frac{1}{8 \log \frac{1-p}{p}}$ and $\varepsilon = \min \left\{ \frac{ \log \frac{1}{2q}}{4(n-1)}, \frac{1+2q}{4q} - 1 \right\}$. Notice that this is a valid choice for $\varepsilon$ since $\frac{1+2q}{4q} - 1 < \frac{1}{2q} -1$. Therefore we know that $q' \le (1+\epsilon )q < \frac{1}{2}$ and we can write:

$$\begin{aligned} \nu&\ge {\overline{d}} \ge \frac{(n-1) (1-q') \log \frac{1-q'}{q'}}{\log \frac{1-p}{p}} > \frac{(n-1) \log \frac{1}{2q'}}{2 \log \frac{1-p}{p}} = \frac{(n-1) \log \frac{1}{2(1+\varepsilon )q}}{2 \log \frac{1-p}{p}} \\&= \frac{(n-1) \log \frac{1}{2q} - (n-1) \log (1+\varepsilon )}{2 \log \frac{1-p}{p}} \ge \frac{(n-1) \log \frac{1}{2q} - \frac{1}{2}\log \frac{1}{2q}}{2 \log \frac{1-p}{p}} \\&= \frac{(n-\frac{3}{2}) \log \frac{1}{2q}}{2 \log \frac{1-p}{p}} \ge \frac{n \log \frac{1}{2q}}{8 \log \frac{1-p}{p}} = \gamma ' n \log \frac{1}{2q}, \end{aligned}$$

where we used the identity $\log (1+x) \le 2x$ for $x \ge 0$. This provides the sought lower bound on $\nu$ and completes the proof. $\square$

## Footnotes

1 In order to ease notation, we omit ceilings in our asymptotic upper bounds. E.g., we will write $O\big ( \frac{n}{k} \log \frac{1}{q}\big )$ in place of $O\big ( \frac{n}{k} \big \lceil \log \frac{1}{q} \big \rceil \big )$. This is only relevant for values of *q* approaching 1.

2 Interestingly, some semiconductor companies consider the more general problem of approximately classifying all their products in a process known as *product binning*. E.g., identically-built processors can be assigned different commercial names depending on their performances.

3 Throughout the rest of the paper we use $\log$ to denote binary logarithms and $\ln$ to refer to natural logarithms.

4 The first parameter of a binomial random variable represents the number of (independent) trials while the second parameter is the success probability of each trial.

5 In order to ease the notation, here and throughout the rest of the paper we will use ${{\textsc {FT}}\hbox {-}{\textsc {Min}}} (\frac{3}{4}n)$ as a shorthand for ${{\textsc {FT}}\hbox {-}{\textsc {Min}}} (\lceil \frac{3}{4}n \rceil )$.

6 Recall from Sect. 4.1 that each query to ${\mathcal {O}}$ is implemented with one comparison when $p \le \frac{1}{16}$, and with $6 c_p + 1$ comparisons when $p > \frac{1}{16}$. Since the number of queries in phase *i* is $2 c_{\mathcal {O}} \Big \lceil \ln \frac{2^i}{q}\Big \rceil + 1 \le 4 c_{\mathcal {O}} \ln \frac{2^i}{q}$, we can pick $\kappa = 4 c_{\mathcal {O}} (6c_p+1)$.

7 The proof of Lemma 4 shows that the probability that more than *i* phases are needed is at most $\left( 5/6 \right) ^i$.

8 If this is not the case, it will suffice to pad *S* with $2^{\lceil \log n \rceil }-n$ randomly selected copies of elements from *S*.

9 The case $k \le \frac{n}{2}$ and $q \in \left( \frac{1}{4}, \frac{1}{2}-\varepsilon \right]$ will be covered by the more general Lemma 11.

10 The lower bound of [14, Theorem 2.1] concerns the related problem of computing the logical OR of an input sequence of bits using noisy queries. A lower bound for this problem immediately translates into a lower bound for ${{\textsc {FT}}\hbox {-}{\textsc {Min}}} (1)$. For details see Lemma 3.4 and Theorem 3.5 in [14] or the self-contained proof of Theorem 4 in Appendix A.

11 The result of a single right rotation on a sequence $\langle x_1, x_2, \dots , x_\ell \rangle$ is the sequence $\langle y_1, y_2, \dots , y_\ell \rangle$ where $y_1 = x_\ell$ and, for $i=2, \dots , \ell$, $y_i = x_{i-1}$.
